# Research advancements in DNA methylation pertaining to ankylosing spondylitis

**DOI:** 10.3389/fimmu.2026.1797758

**Published:** 2026-04-01

**Authors:** Ruibo Xia, Zhihao Qiu, Shuting Kong, Yuhui Ning, Wenhui Zhu, Kepeng Yang

**Affiliations:** 1 The Second Clinical Medical College of Zhejiang Chinese Medical University, Hangzhou, Zhejiang, China; 2 The Second Affiliated Hospital of Zhejiang Chinese Medical University, Hangzhou, Zhejiang, China

**Keywords:** ankylosing spondylitis, biomarkers, bone metabolism, DNA methylation, epigenetics, gene expression, immune regulation

## Abstract

Ankylosing spondylitis (AS) is a persistent autoimmune disorder marked by inflammation of the spine and sacroiliac joints, along with atypical bone development. In recent years, DNA methylation, a significant epigenetic modification, has become a pivotal element affecting AS pathogenesis, disease progression, and clinical diagnosis. This review summarizes what we know so far about abnormal DNA methylation patterns in AS-related genes like DKK1, ERAP1, PDCD1, FOXO1/3a, LGR6, and IRF5, as well as how these patterns affect gene expression. It examines the interaction between DNA methylation and essential pathological processes such as inflammatory responses, immune regulation, and bone metabolism. Additionally, the prospective utilization of DNA methylation as a biomarker for the diagnosis and prognosis of AS is analyzed. Finally, we talk about new ways to treat AS that focus on DNA methylation mechanisms. This review seeks to amalgamate recent methylome and transcriptome studies to establish a comprehensive theoretical framework and delineate future research directions aimed at elucidating epigenetic mechanisms in AS and promoting clinical translation.

## 1 Introduction

Ankylosing spondylitis (AS) is a chronic, progressive autoimmune inflammatory disease that primarily targets the axial skeleton, including the spine and sacroiliac joints, frequently resulting in pain, stiffness, and the eventual fusion of vertebral joints (1). AS is a multifactorial disorder resulting from a complex interaction of genetic predisposition and environmental factors; however, despite extensive investigation, its exact etiology remains unclear. The strong heritability of AS is well known, and the human leukocyte antigen -B27(HLA-B27) allele is the most important genetic risk factor found so far (2). Nonetheless, it is significant that a substantial number of AS patients do not possess HLA-B27, suggesting that other genetic and non-genetic factors influence disease susceptibility. Genome-wide association studies have identified several risk loci beyond HLA-B27, such as ERAP1 and IL23R; however, these genetic variants account for only a small portion of the disease heritability (3). The disparity between established genetic risk factors and disease manifestation has generated interest in epigenetic mechanisms that may facilitate gene-environment interactions and regulate gene expression without modifying the DNA sequence (4).

Epigenetics, especially DNA methylation, has become a promising area for understanding the missing heritability and intricate pathogenesis of AS (5). DNA methylation entails the incorporation of methyl groups into cytosine residues, predominantly at CpG dinucleotides, resulting in alterations in chromatin architecture and the regulation of gene expression. Abnormal DNA methylation patterns can interfere with immune tolerance and inflammatory responses, possibly facilitating the onset of autoimmune diseases. In AS, modifications in DNA methylation have been associated with essential immunological pathways, such as T cell receptor signaling and Th17 cell differentiation, which are pivotal in disease pathogenesis. These epigenetic alterations may affect the expression of genes pertinent to immune regulation, inflammation, and bone metabolism, thereby connecting genetic predisposition with environmental factors (6).

Recent research has pinpointed particular DNA methylation anomalies in AS patients, underscoring their potential as diagnostic biomarkers and therapeutic targets. For example, hypomethylation of the B7-H3 gene promoter is linked to its overexpression, which suggests that it plays a role in changing how the immune system works in AS. Likewise, the promoter regions of genes such as RUNX2 and LGR6 exhibit distinct methylation patterns in AS, correlating with modified gene expression and potentially facilitating pathological bone formation and disease advancement. Moreover, alterations in the methylation of immune checkpoint genes such as The programmed cell death protein 1 (PDCD1) have been associated with their reduced expression, suggesting the role of epigenetic regulation in T cell dysfunction in AS (7). These results highlight the complex function of DNA methylation in regulating immune cell activity and osteogenic processes pivotal to AS pathophysiology (8).

In summary, AS is a complicated autoimmune disease that is affected by both genetics and the environment. Epigenetic changes, especially DNA methylation, are very important in how it starts. DNA methylation modifies gene expression patterns that govern immune responses and bone metabolism, facilitating disease initiation and advancement. Recent studies have identified distinct methylation irregularities in AS patients, yielding new understandings of disease mechanisms and pinpointing potential biomarkers and therapeutic targets. This review systematically summarizes the current progress in understanding DNA methylation in AS, encompassing genetic and molecular mechanisms, clinical applications, and future perspectives, aiming to facilitate the development of innovative diagnostic and treatment strategies for this challenging disorder.

## 2 Abnormalities in DNA methylation of as-related key genes

### 2.1 DKK1 gene methylation and bone lesions

Dickkopf-1 (DKK1) is an important controller of the Wnt signaling pathway, which is very important for making and changing bones. In AS, a chronic inflammatory condition marked by abnormal new bone formation and bone lesions, the epigenetic regulation of DKK1 has attracted considerable interest. Recent studies have shown that the promoter region of the DKK1 gene shows a lot less methylation in people with AS than in healthy controls (6). This means that the levels of DNA methylation may be lower, which could affect gene expression and the development of the disease. This hypomethylation status is linked to lower DKK1 expression, which fits with what we already know about DKK1’s role in stopping Wnt-mediated osteogenesis. The reduced expression of DKK1 resulting from promoter hypomethylation likely plays a role in the increased osteoblastic activity and abnormal bone formation seen in AS.

Additionally, the hypomethylation of DKK1 is significantly associated with inflammatory processes in AS. Research indicates a substantial negative correlation between DKK1 methylation levels and systemic inflammation markers, including C-reactive protein (CRP), neutrophil-to-lymphocyte ratio, and platelet-to-lymphocyte ratio (6). These inflammatory indices are frequently employed to evaluate disease activity, indicating that DKK1 methylation status not only signifies epigenetic modifications but also reflects the inflammatory environment in AS patients. This relationship highlights the potential of DKK1 methylation as a biomarker for disease activity, connecting epigenetic changes to clinical signs.

The methylation status of DKK1 in serum and joint capsule tissues, alongside peripheral blood mononuclear cells, has been correlated with the severity of bone lesions in AS. Lower levels of methylation are linked to more severe bone problems, which suggests that DKK1 methylation could be a way to predict how bone disease will get worse. The capacity to forecast the severity of bone lesions via epigenetic markers such as DKK1 methylation offers potential for enhancing clinical management and customizing therapeutic interventions to alleviate pathological bone formation.

Additionally, the diagnostic utility of DKK1 methylation has been assessed through receiver operating characteristic (ROC) curve analysis, indicating that DKK1 methylation levels may function as a dependable biomarker for AS susceptibility. This discovery is especially significant due to the difficulties in the early diagnosis and monitoring of AS, where traditional imaging and clinical criteria may exhibit insufficient sensitivity during the initial phases. Integrating DKK1 methylation profiling into clinical practice may improve early detection and elucidate disease mechanisms, ultimately informing personalized treatment strategies (9, 10).

In conclusion, the hypomethylation of the DKK1 gene promoter in patients with AS is closely associated with reduced gene expression, increased inflammatory activity, and the severity of bone lesions. These epigenetic alterations not only play a role in the pathophysiology of AS but also present significant diagnostic and prognostic opportunities. Subsequent research ought to concentrate on corroborating these results in more extensive cohorts and investigating the therapeutic alteration of DKK1 methylation as an innovative strategy for addressing bone pathology in AS.

### 2.2 ERAP1 gene methylation and immune regulation

The endoplasmic reticulum aminopeptidase 1 (ERAP1) gene is essential for antigen processing, as it trims peptides for display on major histocompatibility complex class I molecules, thereby affecting immune responses. In AS, a persistent immune-mediated rheumatic disorder, abnormal methylation of the ERAP1 promoter region has been associated with disease pathogenesis. Targeted bisulfite sequencing studies have shown that two CpG islands in the ERAP1 promoter are much more methylated in AS patients than in healthy controls. Quantitative real-time PCR analyses have shown that this hypermethylation is linked to a big drop in ERAP1 mRNA expression (11). The hypermethylation of the promoter that stops ERAP1 expression probably makes it harder for antigens to be processed, which could change how peptides are presented and change immune responses, which could lead to the chronic inflammation that is a hallmark of AS. Furthermore, the methylation status of ERAP1 serves as a molecular hallmark and is associated with clinical parameters, including family history of AS, radiographic severity, and the utilization of non-steroidal anti-inflammatory drugs (NSAIDs). These associations indicate that the epigenetic regulation of ERAP1 is connected to genetic predisposition as well as environmental or therapeutic factors, thereby underscoring its significance in the multifactorial etiology of AS. ERAP1 methylation-induced immune dysregulation may affect the equilibrium of proinflammatory cytokines and T cell subsets, particularly the bias towards Th1/Th17 phenotypes, which are critical in the pathogenesis of AS. Multi-omics Mendelian randomization studies reinforce ERAP1’s significance as a crucial gene in immune response modulation and designate it as a prospective therapeutic target, with protein-level analyses correlating heightened ERAP1 expression to an increased risk of AS (3). Nevertheless, the paradox of hypermethylation resulting in reduced mRNA yet elevated protein levels in certain contexts indicates intricate post-transcriptional regulation that requires additional examination. These findings collectively highlight the importance of ERAP1 promoter methylation in regulating immune function and its prospective role as a biomarker for diagnosis and a target for epigenetic therapies in AS. Ongoing investigation into the mechanistic pathways linking ERAP1 methylation to immune dysregulation and clinical manifestations is crucial for the advancement of precision medicine strategies in the management of AS (12).

### 2.3 PDCD1 gene methylation and t cell function

The programmed cell death protein 1, which is made by the PDCD1 gene, is an important immune checkpoint receptor that controls T cell activity to keep the immune system in balance and stop autoimmunity. Epigenetic regulation, notably DNA methylation of the PDCD1 promoter, is crucial in modulating PDCD1 expression, thereby affecting T cell functionality (13, 14). Elevated methylation levels in the PDCD1 promoter region correlate with diminished mRNA expression of PDCD1, potentially compromising the immunosuppressive function of T cells. This epigenetic repression constrains PDCD1 surface expression, potentially diminishing the inhibitory signaling that typically suppresses T cell activation and proliferation. On the other hand, losing PDCD1 promoter methylation is linked to higher PDCD1 levels. This is seen in CD8+ T cells that are tired from chronic viral infections, where demethylation makes PDCD1 levels stay high and T cells stop working properly. Moreover, DNA methylation regulates the transcriptional activity of PDCD1 in human CD4+ T helper cell subsets, suggesting that methylation patterns influence the functional plasticity and effector differentiation of T cells. The methylation status of PDCD1 serves as an indicator of T cell exhaustion and is associated with systemic inflammatory markers, including ESR and CRP, in addition to disease activity scores such as the AS Disease Activity Score (ASDAS). These correlations indicate that PDCD1 methylation signifies persistent immune activation and inflammation in autoimmune disorders, irrespective of HLA-B27 status, a recognized genetic risk factor for AS. Epigenetic regulators like CBX4 have been demonstrated to inhibit Pdcd1 expression by facilitating repressive histone modifications at its promoter (15), underscoring a complex interaction between DNA methylation and histone modifications in the regulation of PDCD1 expression. Cytokine signaling pathways, particularly the IL-6/STAT3 axis, can counteract epigenetic repression and promote Pdcd1 transcription, thereby influencing T cell exhaustion and immune responses. Clinically, modified PDCD1 methylation and expression patterns have been associated with tumor immune evasion, chronic infections, and autoimmune diseases, highlighting their potential as biomarkers for disease activity and therapeutic targets (16, 17).

To sum up, PDCD1 promoter hypermethylation lowers PDCD1 mRNA expression, which makes T cells less able to suppress the immune system. On the other hand, hypomethylation raises PDCD1 levels, which is linked to T cell exhaustion. The methylation level of PDCD1 shows a positive correlation with inflammation markers and disease activity in AS, regardless of HLA-B27 genotype. This underscores its importance in regulating T cell-mediated immune responses in inflammatory diseases.

### 2.4 FOXO family gene methylation and expression changes

The Forkhead box O (FOXO) family of transcription factors, especially FOXO1 and FOXO3a, is very important for controlling cellular processes like growth, homeostasis, metabolism, and immune responses. Recent research has underscored notable epigenetic alterations in these genes concerning AS, an autoimmune inflammatory disorder marked by persistent inflammation and abnormal bone remodeling. A targeted examination of the methylation status of the FOXO1 gene promoter demonstrated significant hypermethylation in various CpG islands and sites in AS patients relative to healthy controls. This epigenetic modification was associated with a significant reduction in FOXO1 mRNA expression, demonstrating that promoter methylation directly inhibits gene transcription. The negative correlation between methylation levels and mRNA expression (r = -0.624, p < 0.001) highlights the regulatory influence of DNA methylation on FOXO1 gene activity in the pathogenesis of AS (18).

Additionally, subgroup analyses revealed that male AS patients and individuals positive for HLA-B27—a genetic marker significantly correlated with AS—showed elevated FOXO1 promoter methylation levels, indicating a potential interplay between genetic predisposition and epigenetic regulation. Clinically, the level of FOXO1 hypermethylation exhibited a positive correlation with disease activity scores, including the ASDAS (18), indicating its potential as a biomarker for disease severity. On the other hand, methylation levels were negatively correlated with lymphocyte counts, monocyte counts, red cell distribution width, and disease duration. This shows that there is a complicated relationship between the epigenetic status of FOXO1, the movement of immune cells, and the chronicity of AS.

The functional consequences of modified FOXO1 expression are significant, considering FOXO1’s recognized functions in immune regulation and bone metabolism. FOXO1 regulates T cell homeostasis and the production of inflammatory cytokines, both of which are essential in the autoimmune cascade of AS. FOXO transcription factors also affect the differentiation of osteoblasts and osteoclasts, which in turn affects the bone remodeling processes that are out of whack in AS (19). The observed methylation-induced downregulation of FOXO1 may contribute to the dysregulation of immune responses and abnormal bone formation characteristic of AS, establishing a connection between epigenetic modifications and both immunopathology and skeletal manifestations.

While direct data on FOXO3a methylation in AS are scarce, its strong functional association with FOXO1 indicates the potential involvement of analogous epigenetic mechanisms. The simultaneous dysregulation of FOXO family members through DNA methylation may constitute a pivotal mechanism in AS pathogenesis, linking immune dysregulation with metabolic and skeletal irregularities. These findings enhance the comprehension of AS molecular mechanisms and underscore FOXO1 methylation as a potential diagnostic marker and therapeutic target. Interventions designed to modify FOXO1 methylation status or reinstate its expression may mitigate disease activity and avert structural damage in AS patients, necessitating further investigation into epigenetic therapies in this domain.

### 2.5 Epigenetic regulation of LGR6 and IRF5 genes

The epigenetic modulation of genes like leucine rich repeat containing G protein-coupled receptor 6 (LGR6) and IRF5 has become a crucial focus in elucidating the molecular mechanisms associated with AS.Interferon regulatory factor 5 (LGR6), a member of the leucine-rich repeat-containing G protein-coupled receptor family, has been identified as displaying significant hypomethylation correlated with reduced expression levels in patients with AS (20). This hypomethylation indicates a possible disruption of LGR6’s normal function, which may play a role in the development of AS. While direct investigations of LGR6 in AS are scarce, the identified epigenetic modifications suggest that LGR6 may influence cellular signaling pathways associated with inflammation and tissue remodeling, which are essential processes in the pathogenesis of AS. Lower levels of LGR6 expression caused by epigenetic changes could make it less able to control things, which could make inflammatory cascades worse or change how immune cells work. This hypothesis corresponds with extensive evidence indicating that epigenetic modifications can affect gene expression patterns associated with autoimmune and inflammatory diseases.

The IRF5 gene, which encodes interferon regulatory factor 5, is another important player whose epigenetic state is linked to AS. The promoter region of IRF5 exhibits hypomethylation in AS patients, which is associated with increased mRNA expression levels. IRF5 is a well-known transcription factor that controls pro-inflammatory cytokines and the body’s natural immune response. The increased expression caused by promoter hypomethylation is likely what makes AS more inflamed (21). The upregulation of IRF5 can increase the production of cytokines like TNF-α and IL-6, which are very important for the disease to get worse and worse. This epigenetic deregulation highlights a mechanism that sustains immune dysregulation at the transcriptional level in AS. Furthermore, IRF5’s participation in the differentiation and functionality of immune cells reinforces its role in regulating the chronic inflammation seen in AS patients.

The epigenetic changes in LGR6 and IRF5 together show how DNA methylation and gene expression work together in the development of AS. Hypomethylation of these genes may function as a biomarker for disease activity and a prospective therapeutic target. Current studies have not completely clarified the downstream consequences of LGR6 hypomethylation; however, the association between IRF5 promoter hypomethylation and heightened inflammatory gene expression is more clearly defined, underscoring the significance of epigenetic regulation in immune-mediated disorders. Future investigations centered on these epigenetic modifications may yield profound insights into the molecular pathways that underpin AS and facilitate the development of innovative diagnostic and therapeutic approaches aimed at reversing abnormal DNA methylation patterns. This approach is in line with the growing understanding that focusing on epigenetic changes could help treat complicated autoimmune diseases like AS.

### 2.6 Other relevant genes and methylation features

Recent progress in epigenetic research has revealed a complex array of DNA methylation modifications affecting numerous genes beyond the traditional susceptibility loci in AS. Among these, the hypomethylation of the B7-H3 gene promoter has been recognized as a significant epigenetic alteration resulting in its overexpression, which subsequently facilitates immune activation. A case-control study in an eastern Chinese Han population revealed that the B7-H3 promoter region shows significant hypomethylation in AS patients relative to healthy controls, which correlates with elevated mRNA expression levels of B7-H3 in peripheral blood samples. This epigenetic deregulation indicates that the overexpression of B7-H3, induced by promoter hypomethylation, may play a role in the abnormal immune responses associated with AS pathogenesis, establishing B7-H3 as a potential biomarker and therapeutic target (22).

In addition to individual gene studies, genome-wide methylation profiling has yielded comprehensive insights into the epigenetic dysregulation of T cell-associated signaling pathways in AS. Integrative epigenome-wide association studies, alongside transcriptomic analyses of peripheral blood mononuclear cells (PBMCs) from AS patients, identified thousands of differentially methylated positions (DMPs), a substantial number of which were corroborated in independent cohorts. These methylation changes were particularly prevalent in genes associated with T cell receptor (TCR) signaling and T helper 17 (Th17) cell differentiation pathways. Essential genes, including LCK, FYN, CD3G, TCF7, ZAP70, CXCL12, and PLCG1, constituted interconnected regulatory networks wherein DNA methylation levels exhibited a significant correlation with mRNA expression (23). This highlights the critical role of epigenetic regulation in influencing T cell function and inflammatory responses in AS. The research also underscored heightened DNA methylation variability in AS patients, indicating epigenetic heterogeneity that may affect disease phenotypes.

**Figure 1:**
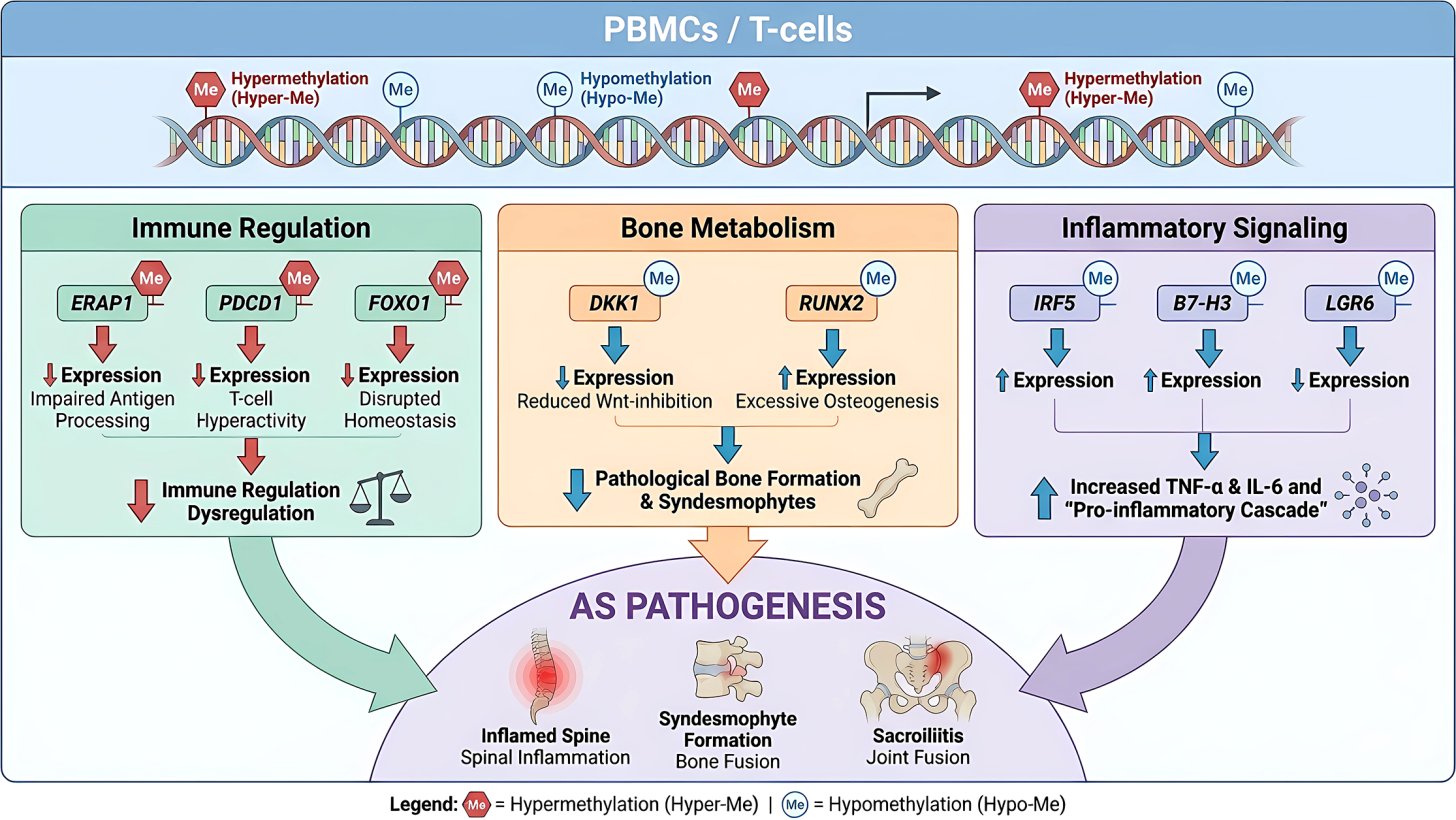
Schematic overview of DNA methylation-mediated pathogenesis in ankylosing spondylitis.

Multi-gene methylation and transcriptional correlation networks add to the complexity of AS epigenetics by showing complicated regulatory axes that affect how the disease develops and how likely it is to happen. For instance, changes in DNA methylation in genes like ERAP1, FOXO1, FOXO3a, LGR6, IRF5, RUNX2, and DKK1 have been linked to AS on their own through unusual promoter methylation patterns that are linked to changes in gene expression and clinical parameters. DNA methylation abnormalities in AS-associated genes are summarized in **Table 1**. These genes are involved in a variety of biological processes, such as processing antigens, regulating transcription, signaling the immune system, remodeling bones, and triggering inflammation. The combination of methylation and transcriptomic data has revealed cis-acting methylation-expression relationships that might play a role in the development of AS. These multi-gene epigenetic networks highlight the intricacy of AS regulation at the epigenomic level and imply that combinatorial epigenetic modifications drive immune dysregulation, inflammation, and aberrant bone formation in AS. A schematic overview of the proposed DNA methylation‑mediated pathogenesis is shown in **Figure 1**. This multilayered epigenetic regulation presents promising opportunities for innovative diagnostic biomarkers and targeted epigenetic therapies.

**Table 1:** Summary of DNA methylation abnormalities in AS-associated genes.

Gene	Methylation status in AS	Effect on gene expression	Key pathways involved	Clinical/pathological significance
DKK1	Hypomethylation	↓Expression	Wnt/β-catenin signaling	Promotes pathological bone formation; correlates with CRP, NLR, and radiographic severity; diagnostic potential
ERAP1	Hypermethylation	↓mRNA expression	Antigen processing/MHC class I presentation	Associated with family history, NSAID use, radiographic severity; influences immune regulation
PDCD1	Hypermethylation	↓Expression	Immune checkpoint/T cell inhibition	Reduces T cell suppression; correlates with ESR, CRP, ASDAS; independent of HLA-B27 status
FOXO1	Hypermethylation	↓Expression	Immune metabolism, osteoblast/osteoclast differentiation	Correlates with ASDAS; inversely correlates with lymphocyte counts; male/HLA-B27+ patients show higher methylation
FOXO3a	Hypermethylation	↓Expression	Immune regulation, bone remodeling	Correlates with ESR, CRP, ASDAS; potential prognostic marker
LGR6	Hypomethylation	↓Expression	G-protein coupled receptor signaling	Moderate diagnostic accuracy; gender-specific methylation variations
IRF5	Hypomethylation	↑Expression	Pro-inflammatory cytokine production (TNF-α, IL-6)	Drives chronic inflammation; contributes to immune dysregulation
B7-H3	Hypomethylation	↑Expression	Immune activation	Promotes abnormal immune responses; potential therapeutic target
miR-495	Hypermethylation	↓miRNA expression	PDCD10 targeting	High diagnostic sensitivity/specificity; negative correlation with PDCD10

CRP, C-reactive protein; NLR, neutrophil-to-lymphocyte ratio; ASDAS, Ankylosing Spondylitis Disease Activity Score; ESR, erythrocyte sedimentation rate; NSAID, non-steroidal anti-inflammatory drug.

In summary, recent findings from targeted and genome-wide methylation studies indicate that AS is defined by a series of epigenetic modifications impacting various genes and pathways. The hypomethylation of the B7-H3 promoter illustrates how particular epigenetic modifications can induce immune activation. Simultaneously, genome-wide methylation anomalies in T cell-related pathways, including TCR signaling and Th17 differentiation, highlight the pivotal role of epigenetic regulation in immune cell function in AS. The intricate interactions among various differentially methylated genes and their transcriptional outputs indicate a sophisticated epigenetic regulatory network that plays a role in the pathogenesis of AS. These findings collectively enhance our comprehension of the epigenetic mechanisms in AS and establish a basis for the development of epigenetic biomarkers and therapeutic approaches.

## 3 Advancements in research on DNA methylation as diagnostic and prognostic biomarkers for AS

### 3.1 Diagnostic value of single-gene methylation biomarkers

The diagnostic potential of single-gene DNA methylation markers in AS has attracted considerable interest, with multiple genes exhibiting encouraging sensitivity and specificity profiles. Among these, DKK1, ERAP1, PDCD1, FOXO3a, and LGR6 have been emphasized due to their abnormal methylation patterns associated with AS diagnosis (1, 24). For example, DKK1, a known modulator of the Wnt signaling pathway involved in bone remodeling, shows significant hypomethylation in AS patients compared to healthy controls. The levels of methylation are inversely related to inflammatory markers like CRP and the neutrophil-to-lymphocyte ratio, which suggests that it plays a role in disease susceptibility and activity monitoring. In a similar way, LGR6, which is a G-protein coupled receptor with leucine-rich repeats, shows significant hypomethylation and lower transcript levels in AS. An analysis of the ROC curve shows an area under the curve of 0.676, which means that it is moderately accurate for diagnosis. The methylation status of microRNA-related genes, including PDCD1 and FOXO3a, plays a role in the pathogenesis of AS and shows potential for diagnostic use, as their epigenetic regulation influences immune cell function and inflammatory responses; however, direct methylation data specific to AS are currently scarce in the literature. Additionally, miR-495, characterized by hypermethylation in its promoter region in AS (24), is downregulated in patients and exhibits a negative correlation with PDCD10 expression, functioning as a highly specific and sensitive biomarker for AS diagnosis, demonstrated by a robust ROC curve performance. These results highlight the value of single-gene methylation markers in indicating disease presence and elucidating underlying pathogenic mechanisms.

In addition to individual genes, the combination of multiple methylation markers has shown improved diagnostic efficacy. ROC curve analyses utilizing multi-gene methylation panels demonstrate enhanced sensitivity and specificity in comparison to individual markers (25, 26). For instance, methylation profiling studies in autoimmune rheumatic diseases have employed machine learning algorithms to amalgamate methylation data from multiple loci, attaining AUC values surpassing 0.9 in differentiating rheumatoid arthritis subtypes, which exhibit overlapping clinical characteristics with AS. While direct multi-gene methylation panels specifically validated in AS cohorts are still being developed, the underlying principle is corroborated by evidence from analogous diseases and individual gene studies in AS. The integration of methylation levels from genes like DKK1, LGR6, and miR-495, in conjunction with other epigenetic markers, shows potential for the creation of reliable, non-invasive diagnostic instruments with elevated precision. This multi-marker method takes into account the differences between AS and other types of inflammatory arthritis, which makes it easier to tell them apart.

In conclusion, single-gene methylation biomarkers such as DKK1, LGR6, and miR-495 show significant changes in AS patients that are linked to how likely they are to get the disease and how inflamed they are. These biomarkers are also very useful for diagnosing the disease. ROC curve analyses indicate the diagnostic potential of these markers, with multi-gene methylation combinations augmenting diagnostic precision. Ongoing research that incorporates these epigenetic markers into composite panels, validated in extensive AS cohorts, is crucial for translating these findings into clinical practice, providing non-invasive, sensitive, and specific diagnostic methods for early AS detection and disease monitoring.

### 3.2 Correlation between methylation and disease activity and clinical subtypes

Changes in DNA methylation have become important epigenetic markers linked to disease activity and clinical phenotypes in AS. Numerous studies have indicated that abnormal methylation levels of critical genes are associated with established disease activity indices, including the ASDAS and the Bath AS Disease Activity Index as well as with clinical manifestations and radiographic progression. For example, hypermethylation of the ERAP1 gene promoter is strongly linked to AS susceptibility and is linked to clinical factors like family history, NSAID use, X-ray classification, and disease symptoms. This suggests that it may be useful for figuring out how severe the disease is and how active it is. Hypomethylation of the FOXO3a promoter CpG islands is inversely associated with inflammatory markers, including erythrocyte sedimentation rate, CRP, and ASDAS (1). Additionally, FOXO3a mRNA levels are diminished in AS patients, further establishing a connection between methylation status and disease activity. The PDCD1 gene promoter is hypermethylated in AS patients, with methylation levels positively correlating with ESR, CRP, and ASDAS scores, and inversely related to gene expression, suggesting that epigenetic regulation of immune checkpoints affects clinical severity. Additionally, alterations in the methylation of the DKK1 gene, which plays a role in bone remodeling, exhibit a negative correlation with inflammatory markers and radiographic progression, underscoring the significance of methylation status in forecasting disease stage and prognosis. The methylation profile of LGR6 demonstrates hypomethylation in AS patients with diminished transcript levels, and subgroup analyses reveal gender-specific methylation variations that may affect clinical phenotypes. These epigenetic modifications not only indicate disease activity but also possess diagnostic potential, as ROC analyses of methylation markers, including ERAP1, FOXO3a, PDCD1, and LGR6, exhibit moderate to high sensitivity and specificity in differentiating AS patients from healthy controls. Moreover, integrative analyses that combine methylome and transcriptome data show that changes in DNA methylation affect gene expression networks that are important for T cell receptor signaling and Th17 differentiation pathways, which are important for the development of AS and its clinical variability. These findings collectively highlight the strong correlation between DNA methylation status and disease activity scores as well as clinical subtypes in AS, providing significant biomarkers for disease staging, prognosis, and tailored therapeutic approaches. Subsequent investigations ought to concentrate on longitudinal studies to authenticate methylation markers as indicators of disease progression and treatment response, thereby improving the clinical management of AS patients.

### 3.3 Clinical application challenges of methylation biomarkers

The clinical application of DNA methylation biomarkers in AS and associated autoimmune rheumatic diseases encounters considerable challenges, especially regarding the standardization and cost-effectiveness of methylation detection techniques. Targeted bisulfite sequencing and Methyl Target sequencing are two current methylation analysis technologies that are very good at finding changes in locus-specific methylation. Studies that looked at genes like DKK1, LGR6, and HIPK3 in AS patients showed this. Nonetheless, these methodologies necessitate advanced laboratory infrastructure, proficient personnel, and intricate bioinformatics pipelines, thereby constraining their extensive implementation in standard clinical environments. It is even harder to reproduce results in different labs and clinical centers because there are no standardized protocols for sample collection, DNA extraction, bisulfite conversion, and sequencing. For example, differences in the efficiency of bisulfite conversion or the depth of sequencing can cause methylation quantification to be inconsistent, which can hurt the accuracy of diagnoses (27). The cost of targeted methylation sequencing is still quite high compared to regular serological tests, which makes it hard to do large-scale screening or long-term monitoring. Machine learning models have been utilized to enhance diagnostic performance through methylation data, exemplified by the AIM2 and CXCR5 methylation studies for rheumatoid arthritis (25, 26). However, the incorporation of these computational tools into clinical workflows necessitates additional validation and resource allocation. To make it easier to use in clinical settings, there is a strong need for cost-effective, high-throughput, and standardized methylation detection platforms that can be used in a wide range of healthcare settings. Simplified assays, including methylation-specific PCR or digital droplet PCR aimed at validated methylation sites, may present viable alternatives; however, they necessitate stringent validation to guarantee sensitivity and specificity on par with sequencing-based techniques. To turn promising methylation biomarkers into reliable clinical tests and personalized therapeutic monitoring tools for AS and other autoimmune diseases, we need to overcome these technical and economic problems.

In addition to technical standardization, the clinical applicability of methylation biomarkers in AS is limited by the restricted scale and scope of current studies, highlighting the necessity for comprehensive multicenter validation and longitudinal follow-up. Most current studies include relatively small cohorts, frequently sourced from a single center, which limits the generalizability of results due to potential population heterogeneity and sampling bias. For instance, research indicating hypomethylation of genes like DKK1, LGR6, and PRICKLE1 in patients with AS has demonstrated encouraging diagnostic potential, evidenced by moderate area under the curve values in ROC analyses (28). Nonetheless, these results necessitate validation in larger, ethnically heterogeneous cohorts to determine reliable diagnostic thresholds and evaluate inter-individual variability. Furthermore, autoimmune rheumatic diseases demonstrate variable disease trajectories affected by therapeutic and environmental factors, thereby requiring longitudinal studies to assess the temporal stability of methylation markers and their association with disease activity, progression, and therapeutic response. Long-term follow-up data are crucial to ascertain whether methylation changes precede clinical manifestations, thereby acting as early predictive biomarkers, or if they signify secondary epigenetic alterations resulting from inflammation. Moreover, multicenter studies facilitate the evaluation of confounding variables, including comorbidities, medication usage, and demographic factors, that may influence methylation patterns. The combination of methylation biomarkers with clinical parameters and other molecular data using advanced statistical and machine learning models has been shown to enhance diagnostic accuracy in rheumatoid arthritis and may be relevant to AS. Nonetheless, these integrative methodologies necessitate validation in extensive cohorts utilizing standardized data collection protocols. In conclusion, the incorporation of methylation biomarkers into clinical practice for AS depends on extensive multicenter studies with prolonged follow-up to confirm their diagnostic, prognostic, and therapeutic monitoring efficacy, thereby guaranteeing clinical reliability and applicability.

### 3.4 Inherent biological and methodological challenges

In addition to technical and financial obstacles, the clinical application of DNA methylation biomarkers in AS faces considerable biological and methodological challenges.Cellular heterogeneity is a major biological concern. PBMCs, which are often used as samples, are a mix of different types of immune cells. As a result, changes in the relative amounts of different cell types (like lymphocytes and monocytes) can make bulk methylation profiles less accurate. They don’t show true cell-intrinsic epigenetic reprogramming in disease-relevant subsets. This phenomenon is illustrated in rheumatoid arthritis, where circulating methylation biomarkers frequently indicate alterations in immune cell composition rather than functional epigenetic modifications within particular pathogenic lineages such as Th17 or regulatory T cells (29).

Methodologically, the discipline is hindered by the predominant dependence on cross-sectional study designs, which significantly restrict causal inference. It is still unclear whether the observed differential methylation is a primary factor in the pathogenesis of AS or merely a secondary effect of chronic inflammation, pharmacological treatment, or other environmental exposures (30). Differentiating correlation from causation constitutes a primary challenge in epigenetic research (31); specific methylation signatures may merely be ‘epiphenomena’ associated with the disease state, lacking representation as actionable nodes within pathogenic pathways.

Adding to these problems is the very important problem of tissue specificity. The principal pathology of AS is located in the axial skeleton and entheses; however, the majority of biomarker studies depend on the readily obtainable peripheral blood. It is still mostly unknown how well blood-based methylation patterns reflect the epigenetic landscape in inflamed joint or spinal tissue. There is not much direct evidence linking blood methylation signatures to the progression of AS seen in X-rays or histopathology. This makes these markers less useful for accurately showing local disease activity or predicting structural damage (32).

To deal with these linked problems, a multi-pronged approach is needed. First, prospective longitudinal cohort studies are necessary to transcend mere associations and to establish temporal relationships and causality. Second, intervention-based experimental methodologies in model systems are essential for evaluating mechanistic hypotheses. Lastly, it is very important to confirm the tissue concordance of suggested biomarkers. New strategies, like looking at the methylation profiles of cell-free DNA to find tissue-of-origin signatures, could be a way to connect peripheral signals and local pathology. However, this needs to be tested specifically in spondyloarthritis (33).

## 4 Potential association between DNA methylation and AS treatment

### 4.1 Methylation modifications affect drug response

Epigenetic modifications, especially DNA methylation, have become significant regulators of gene expression that may affect drug response in AS (34). ERAP1 (endoplasmic reticulum aminopeptidase 1) is one of the most important genes that is involved in the development of AS and how well treatments work. Multi-omics Mendelian randomization studies have pinpointed ERAP1 as a gene exhibiting modified methylation and expression profiles linked to AS susceptibility and severity, indicating that its epigenetic regulation may influence disease activity and therapeutic outcomes. ERAP1 is very important for processing and presenting antigens, which affects immune responses that are very important to the pathophysiology of AS. The methylation status of ERAP1 may influence its expression and protein function, potentially impacting the effectiveness of NSAIDs, which are frequently employed as first-line treatment in AS. While direct clinical evidence connecting ERAP1 methylation to NSAID response is scarce, the correlation of ERAP1 methylation with AS risk and immune regulation suggests that epigenetic modifications may influence inter-individual variability in NSAID efficacy (35).

Likewise, the methylation of absent in melanoma 2 (AIM2), a gene implicated in inflammasome activation and the production of inflammatory cytokines, has been associated with varying responses to anti-TNF-α therapy in patients with AS. Anti-TNF-α agents are essential in the management of AS, especially for patients who do not respond adequately to NSAIDs. Epigenetic regulation of AIM2 may impact the inflammatory environment by altering inflammasome activity (36), consequently influencing the therapeutic effectiveness of TNF-α inhibitors. While particular methylation patterns of AIM2 associated with treatment outcomes necessitate additional validation, nascent evidence endorses the notion that DNA methylation profiles may function as biomarkers for forecasting responses to biologic therapies in AS (37).

In general, these results show that epigenetic changes, like DNA methylation of ERAP1 and AIM2, could affect how drugs work in AS. Combining methylation data with genetic and proteomic data is a promising way to find new therapeutic targets and tailor treatment plans to each patient. By clarifying the molecular mechanisms by which methylation affects gene expression and immune function, forthcoming research may facilitate the creation of epigenetic biomarkers to forecast drug responsiveness and inform precision medicine strategies in the management of AS. This strategy corresponds with the overarching trend of utilizing multi-omics analyses to enhance therapeutic interventions and elevate clinical outcomes in autoimmune diseases.

### 4.2 Prospects and challenges of epigenetic-targeted therapy

Epigenetic-targeted therapies, especially those that focus on changing DNA methylation, are becoming promising options for treating AS. This is a new approach that goes beyond traditional immunosuppressive or biologic agents. DNA methyltransferase inhibitors, capable of reversing abnormal DNA methylation patterns, have demonstrated initial efficacy in modulating immune responses associated with AS pathogenesis. The combined study of methylome and transcriptome data in AS patients shows that DNA methylation is not working properly at important immune regulatory genes that are involved in Th17 cell differentiation and TCR signaling pathways. These genes include LCK, FYN, and ZAP70, which are important for the inflammatory processes that cause AS. These results indicate that targeting DNA methylation machinery may rectify immune dysregulation by reinstating normal gene expression profiles. Furthermore, particular hypomethylation events in the promoters of genes such as B7-H3 and RUNX2 are associated with their overexpression, which contributes to the inflammatory and osteogenic processes characteristic of AS. Consequently, epigenetic drugs that rectify these methylation anomalies may mitigate disease activity and pathological bone formation. The reversibility of DNA methylation marks provides a therapeutic opportunity to dynamically regulate gene expression, which is especially appealing in chronic autoimmune disorders where immune cell plasticity is essential (38).

The emergence of methylation biomarkers enhances the application of personalized medicine strategies in AS. Methylation signatures of genes like IRF5 and LGR6 have shown promise as diagnostic and prognostic tools, as they are linked to disease severity and inflammatory markers. This shows how useful they are for grouping patients. Integrating methylation profiling into clinical practice enables the identification of patient subgroups that may derive optimal benefit from epigenetic therapies or customized combination regimens tailored to their distinct epigenomic landscape. This model of precision medicine could make treatments more effective while reducing side effects. Moreover, the interaction between DNA methylation and various epigenetic mechanisms, such as non-coding RNAs and histone modifications, can be harnessed to create combinatorial epigenetic therapies that simultaneously target multiple regulatory layers, potentially improving treatment efficacy (39, 40).

Even though these are good signs, there are still some big problems that need to be fixed before epigenetic therapies can be used regularly in AS. At the tool level, current DNMT inhibitors lack adequate specificity, posing a significant risk of off-target effects that may interfere with normal gene expression in non-pathogenic cells (41). This highlights the pressing necessity for more selective agents or targeted delivery systems. At the target level, the significant variation in methylation patterns among distinct immune cell types and disease stages requires comprehensive, cell-specific epigenomic mapping to identify the most therapeutically relevant targets (42). Giving non-specific epigenetic modulators through the whole body may not affect the target pathogenic cell populations and may instead affect bystander cells. At the evidence level, there exists a significant deficiency in clinical validation: thus far, no clinical trials have explicitly evaluated the safety and efficacy of epigenetic modulators in AS (43). Right now, the field relies on preclinical models, like immune cells from patients and animals, to figure out how things work and see how well they might work as treatments. To turn epigenetic insights into safe and effective treatments for people with AS, it will be important to fill this gap in evidence through careful preclinical modeling and, eventually, well-designed clinical trials.

### 4.3 Methylation and regulation of stem cell function

The control of stem cell activity via epigenetic mechanisms, especially DNA methylation, has become a crucial field of study with major consequences for conditions like AS and bone-related disorders. One important molecule is the fat mass and obesity-associated protein (FTO), which changes RNA methylation and has been shown to affect how well mesenchymal stem cells from AS patients can stop osteoclast formation. This discovery indicates that FTO-mediated RNA methylation constitutes a novel therapeutic target for the modulation of bone remodeling in AS. The epigenetic regulation of stem cells, encompassing mesenchymal stem cells and dental-derived mesenchymal stem cells, is essential for preserving their self-renewal and differentiation potential. DNA methylation dynamically regulates gene expression profiles that dictate osteogenesis, adipogenesis, and immunomodulatory functions, thereby affecting stem cell fate and function (44–47).

In the realm of bone disease, abnormal DNA methylation patterns have been associated with pathological conditions such as osteonecrosis of the femoral head, where alterations in methylation influence MSC differentiation, angiogenesis, and osteocyte viability, underscoring the therapeutic potential of targeting DNA methylation to mitigate bone degeneration (48). Additionally, age-associated epigenetic drift in hematopoietic stem and progenitor cells entails alterations in DNA methylation entropy that are associated with stem cell replication rates and functional deterioration, highlighting the significance of methylation homeostasis in stem cell senescence and tissue regeneration.

Mechanistically, DNA methyltransferases (DNMTs), including DNMT3A and DNMT3B, preserve methylation patterns crucial for stem cell commitment and differentiation. For instance, DNMT3A-dependent methylation is essential for spermatogonial stem cells to initiate spermatogenesis; the absence of DNMT3A results in compromised differentiation and abnormal enhancer activation. The ten-eleven translocation (TET) family of enzymes, especially TET2 (45), also controlsprogress. DNA methylation and histone modifications to control HSPC self-renewal and differentiation. When TET2 is missing, aged stem cells lose their ability to function and relocate spatial heterochromatin (47).

Epigenetic regulation encompasses not only DNA methylation but also histone modifications and RNA methylation, which together govern stem cell fate decisions. For example, methylation of arginine in METTL14, which is part of the m6A RNA methyltransferase complex, increases RNA methylation activity, which affects the processes of maintaining stem cells and repairing DNA. The interaction between DNA methylation and histone modifications, specifically methylation at H3K4, H3K9, and H3K27 marks, is essential for the osteogenic and odontogenic differentiation of dental mesenchymal stem cells, influencing tissue regeneration and inflammatory responses. Moreover, the stiffness of the extracellular matrix can influence DNA methylation through protein kinase C α-dependent nuclear transport of DNMT3L, thereby connecting biomechanical signals to the epigenetic regulation of stem cell pluripotency.

Epigenetic mechanisms, particularly those involving DNA and RNA methylation, are increasingly recognized as central to the pathogenesis of ankylosing spondylitis (AS) and related bone disorders. These mechanisms help explain the underlying stem cell dysfunction observed in these conditions and, importantly, reveal promising avenues for therapeutic intervention. By strategically altering the methylation states of key regulatory molecules, it may be possible to restore the critical balance between bone-forming osteogenesis and bone-resorbing osteoclastogenesis. Such a rebalancing could enhance normal bone remodeling processes and mitigate the pathological new bone formation characteristic of AS. The accumulating body of evidence strongly supports the therapeutic potential of targeting specific epigenetic regulators, including FTO, DNMTs, and TET enzymes, to correct stem cell function and develop novel treatments for AS and other skeletal diseases.

To turn promising molecular targets into useful clinical applications, there needs to be a strict and organized preclinical research phase. To fill this gap, strong preclinical models are needed to clearly explain how epigenetic modulators work and find the best times to use them in AS. This basic work usually uses a dual approach, which combines *in vitro* and *in vivo* systems (49).

The initial phase entails mechanistic validation utilizing patient-derived cellular models, including peripheral blood immune cells or mesenchymal stem cells. These systems facilitate regulated hypothesis testing to verify that the alteration of a particular methylation event—such as at regulatory sites like the FOXO1-SIRT1 axis or the B7-H3 promoter—directly results in the desired modifications in gene expression and functional outcomes. For example, one goal is to stop mesenchymal stem cells from differentiating too much into osteogenic cells or to stop pro-inflammatory immune cells from becoming active (50).

After successful *in vitro* validation, the subsequent essential phase involves proof-of-concept studies utilizing appropriate animal models of spondyloarthritis. These *in vivo* models are essential for evaluating the efficacy of candidate epigenetic drugs in mitigating the core pathological characteristics of AS: chronic inflammation and aberrant bone formation (51). They also give a complete biological picture that can be used to look at tissue-specific effects, figure out off-target toxicities, and find the best dosing schedules. The data obtained from these stringent preclinical studies are crucial for mitigating risks in subsequent translational initiatives and informing the design of future human clinical trials, thereby ensuring that first-in-human studies are both scientifically valid and optimally informative (52).

## 5 Problems and future directions in DNA methylation research

### 5.1 Research limitations

Although there is more and more evidence that DNA methylation plays a role in the development of AS, there are still some important problems that make it hard to fully understand and use these findings in the clinic. A significant limitation is the small sample size and the wide range of differences between studies. Most studies use groups of patients and controls that are not very large, usually only a few dozen to a few hundred people. This makes it harder to draw conclusions and use the results in other studies. For instance, investigations analyzing the methylation patterns of genes such as DKK1, ERAP1, PDCD1, FOXO1, FOXO3a, LGR6, and IRF5 frequently involve sample sizes under 100 per group. The cohorts, being relatively small, may not sufficiently represent the genetic, environmental, and clinical diversity present in AS populations, resulting in heterogeneous methylation profiles and inconsistent correlations with clinical parameters such as inflammation markers, disease activity scores, and radiographic progression. Additionally, variations in sample sources, methylation detection methodologies, and data analysis workflows introduce variability and hinder cross-study comparisons. The absence of standardized protocols and cohesive diagnostic or classification criteria for AS intensifies this heterogeneity, hindering reproducibility and the development of consensus methylation biomarkers (34).

Another significant limitation is the insufficient clarification of the causal relationship between alterations in DNA methylation and changes in gene expression in AS. Numerous studies indicate differential methylation of promoter or regulatory regions that correlate with altered mRNA expression levels—such as the hypermethylation of ERAP1 and PDCD1 promoters associated with decreased gene expression, or the hypomethylation of B7-H3 and LGR6 promoters linked to increased expression—these correlations do not conclusively establish causality. Certain studies demonstrate inconsistent or non-significant correlations; for example, DKK1 hypomethylation in AS patients did not align with statistically significant alterations in transcript levels, indicating intricate regulatory mechanisms beyond methylation alone. Furthermore, epigenetic regulation is multifactorial, encompassing interactions among DNA methylation, histone modifications, non-coding RNAs, and chromatin architecture, which remain inadequately represented in contemporary studies. The temporal dynamics of methylation changes in relation to disease onset and progression are not well understood, and it is ambiguous whether methylation alterations are causative factors or effects of inflammation and immune dysregulation in AS. Sophisticated integrative analyses that merge methylome and transcriptome data, alongside causal inference tests, have commenced the identification of potentially causative methylation-expression axes, especially within pathways like Th17 differentiation and T cell receptor signaling. These results necessitate additional validation in larger, longitudinal cohorts and functional experiments to substantiate mechanistic connections.

In summary, the current research on DNA methylation in AS is constrained by small, heterogeneous sample sizes and methodological inconsistencies, alongside an inadequate comprehension of the causal relationship between methylation and gene expression. To reliably find epigenetic biomarkers and therapeutic targets for AS, it will be important to address these limitations through larger, standardized, multi-omics studies and functional validation.

### 5.2 Multi-omics integration analysis

Multi-omics integration signifies a revolutionary methodology for clarifying the intricate epigenetic regulatory networks associated with AS. This approach allows for the creation of complete, multi-layered regulatory networks by systematically combining data from different molecular layers, such as DNA methylation (the epigenome), gene expression (the transcriptome), protein abundance (the proteome), and other epigenetic mechanisms like microRNA (miRNA) activity and histone modifications (53). These networks encompass epigenetic modifications, transcriptional output, and protein function, elucidating the complex interactions between genetic and epigenetic factors in disease pathogenesis. This integrative approach transcends the insights derived from any singular omics layer in isolation, presenting a comprehensive perspective on molecular dysregulation in AS.

A recent multi-omics Mendelian randomization study exemplifies the efficacy of this approach, identifying several pivotal genes—namely TNFRSF1A, B3GNT2, ERAP1, and FCGR2A—that demonstrate consistent and significant associations across methylation, gene expression, and protein levels. This convergent evidence from various omics layers significantly bolsters confidence in the biological relevance of these genes within immune-related inflammatory pathways associated with AS, while also positioning them as promising therapeutic candidates. In addition to these linear correlations, integrative analyses have uncovered disease-specific chromatin states enriched at genetic risk loci, especially in monocytes, underscoring the essential function of epigenetic regulation in immune cells. Researchers have successfully linked functional single nucleotide polymorphisms to their target genes by combining chromatin interaction data with multi-omics profiles. This has given them a better understanding of how genetic variation affects epigenetic regulation and gene expression (54).

Multi-omics approaches are important for showing how DNA methylation works with other epigenetic processes to control gene regulation, beyond just linear correlations between methylation and expression. For example, studies that look at more than one disease have shown that changes in DNA methylation can directly affect the expression of miRNAs. These miRNAs then control their target mRNAs, often creating complex feedback loops that fine-tune how cells respond. In the same way, certain histone modifications, like the repressive mark H3K27me3 and the activating mark H3K4me3, have been shown to interact with DNA methylation patterns in a dynamic way to control gene transcription. This has been seen in situations like the response of cells to stress. While a thorough comprehension of these multi-layered epigenetic interactions in AS is still lacking, the analytical frameworks established in other complex diseases offer a solid basis for future research (55).

The progress of computational methods is a key factor in the integration of multi-omics, with machine learning and artificial intelligence playing a major role. These technologies are uniquely adept at integrating heterogeneous omics datasets, even when sourced from disparate sample collections. Algorithms like INTEND can find links between DNA methylation and gene expression in different experiments. This makes it easier to rebuild regulatory networks, even when there aren’t any matched samples. More advanced frameworks, like graph convolutional networks (GCNs), have been used successfully for multi-omics integration tasks like classifying patients and finding biomarkers. MOGONET and other tools use GCNs to create network graphs of molecular interactions that show how different omics layers interact with each other, such as how methylation and gene expression affect each other. These methods make it possible to capture nonlinear relationships, which helps to more accurately rebuild disease-specific regulatory networks and predict important functional nodes (56).

The main translational value of multi-omics integration is that it can find useful biological information. It greatly helps find biomarkers for diagnosis and prognosis, and it also helps figure out which therapeutic targets are the most important. In oncology, multi-omics analyses have successfully pinpointed epigenetically regulated genes that function as dependable biomarkers and therapeutic targets, underscoring the translational efficacy of these methodologies (57). This model can be used directly on AS: combining DNA methylation with transcriptomic and proteomic data can also show important regulatory nodes that can be changed with drugs. Subsequent molecular docking studies employing multi-omics-identified targets have validated promising candidate drugs, demonstrating that integrated analysis enhances mechanistic comprehension and accelerates the advancement of targeted therapies. However, before these computational tools can be fully integrated into routine clinical workflows, they need to be tested again in separate groups and given their own resources. For epigenetic and multi-omic discoveries to lead to real clinical progress for AS patients, multi-omics integration must continue to be improved and used (58).

### 5.3 Clinical translational research

The clinical application of DNA methylation research in AS represents a nascent domain with considerable potential to enhance diagnosis, prognosis, and individualized treatment approaches (27). One important area is making methylation detection technologies that are cheap, very sensitive, and good for use in clinical settings. Recent research has employed targeted bisulfite sequencing and methylation-specific PCR to measure methylation alterations in genes including DKK1, ERAP1, PDCD1, FOXO1, and LGR6, revealing their abnormal methylation profiles in AS patients relative to healthy controls. Nonetheless, these techniques, although accurate, may be expensive and technically challenging for regular clinical use. Progress in digital PCR, microfluidics, and enzymatic conversion techniques shows promise in addressing these obstacles by facilitating rapid, sensitive, and quantitative methylation detection, even in small or degraded samples like cell-free DNA (59). These technological advancements may enable extensive screening and surveillance of methylation biomarkers in AS, thereby rendering epigenetic assays more accessible and cost-effective.

To determine the diagnostic and prognostic value of methylation biomarkers in AS, large-scale clinical cohort validations are necessary. Several studies have indicated the diagnostic utility of methylation modifications in particular genes. For example, changes in the methylation levels of DKK1 and LGR6 (hypomethylation), ERAP1 and PDCD1 promoters (hypermethylation), and FOXO1 and IRF5 (methylation changes) have been linked to AS susceptibility and disease activity. ROC analyses have shown moderate to high sensitivity and specificity. Furthermore, associations between methylation levels and clinical parameters, including CRP. erythrocyte sedimentation rate, Bath AS Disease Activity Index, and radiographic progression have been reported. Even though these results are promising, most studies have small sample sizes and only look at one center’s cohorts. Consequently, multicenter, longitudinal studies involving larger and more heterogeneous populations are essential to rigorously validate these methylation markers, evaluate their reproducibility, and establish standardized thresholds for clinical decision-making.

The investigation of methylation-based personalized therapeutic strategies signifies an innovative and auspicious approach in the management of AS. Epigenetic alterations, including DNA methylation, affect gene expression patterns that regulate the immune system, inflammation, and bone remodeling, all of which are important to the development of AS. The FOXO1-SIRT1 axis, influenced by methylation status, impacts immune metabolism and osteogenic differentiation, indicating that targeting this pathway may alter disease progression. The discovery of methylation alterations in immune checkpoint genes such as PDCD1 and inflammatory regulators like IRF5 paves the way for epigenetic therapies aimed at reinstating normal immune tolerance and mitigating inflammation. Additionally, the reversibility of DNA methylation marks presents opportunities for therapeutic interventions utilizing DNA methyltransferase inhibitors or demethylating agents, which have demonstrated efficacy in other diseases. Combining methylation profiling with other omics data could help doctors group patients based on their epigenetic signatures. This would make it possible to create personalized treatment plans that work best for each patient while causing the least amount of harm.

In conclusion, clinical translational research in DNA methylation for AS is advancing towards the creation of sensitive, economical methylation assays, the validation of methylation biomarkers in extensive cohorts, and the formulation of methylation-targeted personalized therapies. To fully realize the potential of DNA methylation as a tool for improving AS diagnosis, prognosis, and treatment, we need to keep working on new technologies, rigorous clinical validation, and mechanistic studies.

## 6 Conclusions

In conclusion, DNA methylation has become a crucial epigenetic mechanism intricately linked to the pathogenesis, progression, and clinical manifestations of AS. From an expert standpoint, the amassed evidence highlights the intricacy of AS as a multifactorial disorder, wherein anomalous DNA methylation patterns in critical AS-associated genes, including DKK1, ERAP1, PDCD1, FOXO1/3a, LGR6, and IRF5, profoundly affect gene expression profiles and interfere with immune and bone metabolic pathways. These epigenetic changes not only cause the immune system to work poorly and the bones to remodel in a way that is typical of AS, but they also have a strong link to disease activity, inflammatory markers, and structural bone damage. This underscores the dual function of DNA methylation as both a mechanistic catalyst and a prospective biomarker for disease diagnosis and prognosis.

Taking into account the different research points of view, it is clear that while many studies have found specific methylation changes linked to AS, the results are not always the same because the patient groups, tissue types, and methods used are different. This variability necessitates careful interpretation and underscores the significance of integrating multi-omics data—including genomics, transcriptomics, and proteomics—to develop a comprehensive epigenetic regulatory network. This integrative approach will improve our understanding of how DNA methylation interacts with other molecular layers. This will make AS diagnosis more accurate and allow for the creation of personalized treatment plans.

DNA methylation research has implications that go beyond understanding how things work; it also provides a theoretical basis for precision medicine in AS. By identifying methylation patterns that show how diseases and treatments work, doctors may soon be able to group patients more effectively and change their treatments as needed. Furthermore, the discovery of methylation-based biomarkers facilitates the development of non-invasive diagnostic tools and the monitoring of disease progression, which are essential for enhancing patient outcomes.

To move forward with the clinical translation of DNA methylation findings, we need to work together in a number of areas. Extensive, meticulously structured clinical studies are imperative to authenticate potential methylation markers and evaluate their sensitivity and specificity across varied populations. Furthermore, mechanistic studies that clarify the interactions between methylation and other epigenetic modifications, including histone modifications and non-coding RNAs, will enhance our comprehension of AS pathophysiology. Such knowledge is essential for the creation of innovative epigenetic therapies capable of altering abnormal methylation patterns, potentially reversing disease mechanisms or preventing progression.

In conclusion, the changing field of DNA methylation research in AS shows how genetic predisposition, epigenetic regulation, and environmental factors all work together. By bringing together different research results and using integrative multi-omics methods, the field is ready to discover new aspects of AS biology. This will not only improve the ability to diagnose and predict the disease, but it will also speed up the development of new, targeted treatments that will make life better for people with this debilitating disease.
